# Editorial for Special Issue “Natural Compounds: An Adjuvant Strategy in Cancer Management”

**DOI:** 10.3390/cimb48060544

**Published:** 2026-05-22

**Authors:** Monia Cecati, Arianna Vignini

**Affiliations:** 1 Department of Human Science and Promotion of the Quality of Life, San Raffaele Roma Open University, 00166 Rome, Italy; 2 Department of Clinical Sciences, Università Politecnica delle Marche, 60100 Ancona, Italy

Despite remarkable advances in cancer therapy, treatment resistance, tumor heterogeneity, and therapy-associated toxicity remain major obstacles in achieving durable clinical responses [1]. These challenges have stimulated increasing interest in natural compounds as complementary and adjuvant strategies capable of modulating multiple cancer-related pathways while improving therapeutic tolerability [2,3].

The Special Issue “Natural Compounds: An Adjuvant Strategy in Cancer Management” provides an integrative overview of the anticancer potential of natural products, highlighting their role as multi-target modulators capable of interfering with tumor growth, metastasis, inflammation, and resistance mechanisms. The presented contributions span experimental investigations and comprehensive reviews, collectively supporting the emerging paradigm of combining natural compounds with conventional therapeutic approaches. A prominent theme across the Special Issue is the ability of natural compounds to enhance the efficacy of standard anticancer treatments. Evidence of synergistic interactions is demonstrated by fermented noni extract, which significantly potentiates the cytotoxic effects of chemotherapeutic agents while promoting apoptosis and suppressing tumor cell migration [4]. Similarly, bioactive steroidal saponins derived from *Marsdenia tenacissima* fermentation exhibit targeted modulation of EGFR signaling and apoptotic pathways, suggesting a potential role in overcoming resistance mechanisms in lung cancer [5]. Another recurring mechanistic hallmark is the induction of oxidative stress and apoptosis as central anticancer strategies. Juglone and D-limonene exemplify this mechanism in colorectal cancer models, where reactive oxygen species generation, apoptosis activation, and inflammatory modulation collectively contribute to antiproliferative effects [6,7]. These findings reinforce the concept that redox imbalance represents a critical vulnerability that can be exploited by natural compounds. Beyond oxidative stress, several studies emphasize the multi-target signaling modulation exerted by plant-derived compounds. The identification of saponins from Aralia chinensis highlights their capacity to interfere with key oncogenic pathways, including Src, PI3K, and EGFR signaling, thereby inhibiting breast cancer cell proliferation and survival [8]. This multi-pathway targeting aligns with the complexity of tumor biology and underscores the potential of natural compounds to address pathway redundancy and adaptive resistance. Complementing experimental research, the review articles included in this Special Issue provide a broader translational perspective. Curcumin emerges as a paradigmatic multi-target phytochemical capable of regulating Wnt, PI3K/Akt/mTOR, MAPK, and JAK/STAT pathways while influencing ferroptosis and epigenetic regulation. Importantly, advances in nanoformulation strategies are highlighted as key approaches to overcome bioavailability limitations and enhance clinical applicability [9]. The chemosensitizing potential of plant-derived compounds is further explored through analyses of natural inhibitors of tyrosine kinase signaling, a pathway critically involved in tumor progression and therapeutic resistance, particularly in lung cancer [10]. Additionally, integrative reviews of herbal medicine in breast cancer emphasize the pleiotropic activities of phytochemicals, including apoptosis induction, immune modulation, and tumor microenvironment remodeling [11]. Inflammation-driven carcinogenesis represents another important focus, illustrated by the role of luteolin in the inflammatory bowel disease–colorectal cancer continuum. By modulating inflammatory mediators, oxidative stress, and cell proliferation pathways, luteolin exemplifies the dual anti-inflammatory and anticancer properties characteristic of many natural compounds [12].

Collectively, the contributions of this Special Issue highlight several converging concepts. First, natural compounds display pleiotropic mechanisms that enable simultaneous modulation of multiple hallmarks of cancer. Second, combination strategies with conventional therapies represent a promising approach for enhancing treatment efficacy and mitigating resistance. Third, advances in network pharmacology, systems biology, and nanotechnology are accelerating the identification and translational development of bioactive natural products.

## Future Directions

Future research should prioritize the transition from mechanistic insights to clinically actionable strategies. Standardization of natural compound extracts and rigorous characterization of pharmacokinetics and pharmacodynamics represent critical prerequisites for clinical translation [13,14]. Moreover, the integration of omics-based approaches, artificial intelligence-driven drug discovery, and network pharmacology will likely facilitate the identification of synergistic compound combinations and predictive biomarkers of response. Nanotechnology-based delivery systems represent one of the most promising strategies to improve the pharmacokinetic profile of multi-target phytochemicals, particularly by enhancing solubility, controlled release, and tumor-specific accumulation while minimizing systemic toxicity [15]. Additionally, exploring the immunomodulatory potential of natural compounds in combination with immunotherapy represents a particularly promising avenue, given the emerging evidence linking phytochemicals to tumor microenvironment remodeling [16]. However, the risk of overestimating preclinical efficacy without rigorous clinical validation remains a major concern in the field of natural compounds. Another key research priority involves the design of well-structured clinical trials evaluating natural compounds as adjuvant therapies alongside standard treatments. Precision oncology approaches incorporating patient stratification and biomarker-guided interventions may further enhance therapeutic outcomes [17]. Finally, interdisciplinary collaboration among molecular biologists, pharmacologists, clinicians, and computational scientists will be essential in translating preclinical findings into effective therapeutic strategies. In conclusion, this Special Issue reinforces the growing recognition of natural compounds as valuable adjuvant tools in cancer management. Continued mechanistic exploration, optimization of delivery systems, and rigorous clinical validation will be essential to fully harness their therapeutic potential and translate promising preclinical evidence into effective oncological strategies. As Guest Editors, we extend our sincere gratitude to all authors and peer reviewers for their valuable contributions to this Special Issue and welcome more submissions for its second edition. We also express our gratitude to the editorial staff of the journal for their continuous support throughout the publication process.
